# Analysis of 394 COVID-19 cases infected with Omicron variant in Shenzhen: impact of underlying diseases to patient’s symptoms

**DOI:** 10.1186/s40001-022-00927-1

**Published:** 2022-12-15

**Authors:** Peiyan Zhang, Zhao Cai, Zhiguang He, Peifen Chen, Weibo Wu, Yuanlong Lin, Shiyan Feng, Ling Peng, Jianming Li, Jing Yuan, Liang Yang, Fuxiang Wang, Yingxia Liu, Hongzhou Lu

**Affiliations:** 1 Department of Infectious Diseases, Shenzhen Third People’s Hospital, Second Hospital Affiliated to Southern University of Science and Technology, Shenzhen, 518112 China; 2 School of Medicine, Southern University of Science and Technology, Shenzhen, 518055 China; 3 National Clinical Research Center for Infectious Disease, Shenzhen, 518112 China; 4 Shenzhen Key Laboratory for Gene Regulation and Systems Biology, Southern University of Science and Technology, Shenzhen, 518055 China; 5 Luohu Clinical Institute of Shantou University Medical College, Shantou, China

**Keywords:** SARS-CoV-2, Omicron, IgG antibody, COVID-19 vaccine

## Abstract

**Objectives:**

The emergence of new variants of SARS-CoV-2 is continuously posing pressure to the epidemic prevention and control in China. The Omicron variant of SARS-CoV-2 having stronger infectivity, immune escape ability, and capability causing repetitive infection spread to many countries and regions all over the world including South Africa, United States and United Kingdom etc., in a short time. The outbreaks of Omicron variant also occurred in China. The aim of this study is to understand the epidemiological characteristics of Omicron variant infection in Shenzhen and to provide scientific basis for effective disease control and prevention.

**Methods:**

The clinical data of 394 imported COVID-19 cases infected with Omicron variant from 16 December 2021 to 24 March 2022 admitted to the Third People’s hospital of Shenzhen were collected and analyzed retrospectively. Nucleic acid of SARS-CoV-2 of nasopharyngeal swabs and blood samples was detected using 2019-nCoV nucleic acid detection kit. Differences in Ct values of N gene were compared between mild group and moderate group. The specific IgG antibody was detected using 2019-nCoV IgG antibody detection kit. Statistical analysis was done using SPSS software and graphpad prism.

**Results:**

Patients were categorized into mild group and moderate group according to disease severity. The data on the general conditions, underlying diseases, COVID-19 vaccination and IgG antibody, viral load, laboratory examination results, and duration of hospitalization, etc., were compared among disease groups. Mild gorup had higher IgG level and shorter nucleic acid conversion time. Patients with underlying diseases have 4.6 times higher probability to progress to moderate infection.

**Conclusion:**

In terms of epidemic prevention, immunization coverage should be strengthened in the population with underlying diseases. In medical institutions, more attention needs to be paid to such vulnerable population and prevent further deterioration of the disease.

## Introduction

COVID-19 is a respiratory disease caused by severe acute respiratory coronavirus 2 (SARS-CoV-2), which has become a pandemic and infected millions of people worldwide [1]. Since its onset, SARS-CoV-2 has evolved a series of variants through continuous evolution, such as alpha (B.1.1.7), beta (B.1.351), gamma (P.1), and delta (B.1.617.2) variants [2]. In November 2021, South Africa reported the infected case by Omicron variant for the first time, which had then spread too many countries and regions around the world quickly. As of 20 January 2022, there were 171 countries reported COVID-19 cases infected with Omicron variant which became the dominating epidemic strain in South Africa, the United States and United Kingdom. Since 9 December 2021, China has also been experiencing outbreaks of Omicron infection. Omicron subvariant, BA.2, mainly invades the upper respiratory tract, but some infected patients may also have symptoms of lower respiratory tract infection and pneumonia as indicated by chest CT. Here in this study, we retrospectively collected and compared the differences in clinical conditions, viral load, underlying diseases, vaccination status, level of specific IgG antibody, results of laboratory examinations and hospitalization time among 394 mild and moderate COVID-19 patients infected with Omicron variant admitted to the Third People’s Hospital of Shenzhen. Based on the clinical data, we aim to find out which patients are prone to lower respiratory tract infections and predisposing factors. Clinically, patients with underlying diseases are more prone to pneumonia and need more attention and protection during treatment.

## Materials and methods

### Patients and data collection

394 imported COVID-19 patients infected with Omicron variant from 16 December 2021 to 24 March 2022 admitted to the Third People’s hospital of Shenzhen (the designated hospital for treatment of COVID-19 patients) were enrolled in this study. All patients were diagnosed with COVID-19 according to both the Chinese guidelines for disease diagnosis and treatment and the diagnosis and treatment of COVID-19 in China (trial version 9) [3]. The patients were categorized into mild and moderate group according to the severity of their conditions based on the criteria of the guidelines. Mild case is defined as the cases with mild clinical symptoms without pneumonia symptoms found from imaging. Moderate case is defined as the cases with fever and other respiratory symptoms with pneumonia symptoms from imaging. There were 305 mild cases and 89 moderate cases respectively. Clinical data on their general conditions, underlying diseases, clinical classification, clinical symptoms, vaccination and IgG antibody, laboratory examination results and nucleic acid conversion time were collected retrospectively and compared between the two groups. The patients can only be discharged if fulfilling the following criteria: 1. body temperature within normal range for more than 3 days; 2. respiratory symptoms improved significantly; 3. pulmonary absorption as seen by imaging techniques; 4. Ct values of the nucleic acid tests of respiratory samples ≥ 35 for two consecutive tests with > 24 h interval. Vaccines received by the patients included inactivated viral vaccine, mRNA vaccine, recombinant subunit vaccine and viral vector vaccine.

### Detection of SARS-C0V-2 viral nucleic acid and IgG and IgM antibody

Nasopharyngeal swabs and blood samples were collected while nucleic acid of SARS-CoV-2 was detected by fluorescent PCR method using 2019-nCoV nucleic acid detection kit. Omicron variant was confirmed by whole genome sequencing. The differences in Ct values of N gene were compared among disease groups. The specific IgG antibody was detected using 2019-nCoV IgG antibody detection kit (magnetic particle chemiluminescence). The specific IgM antibody was detected using 2019-nCoV IgM antibody detection kit (magnetic particle chemiluminescence). The differences in the levels of specific IgG/IgM antibody, clinical symptoms, laboratory examination and hospitalization time etc. were also compared among disease groups.

### Statistical analysis

Statistical analysis was performed using SPSS software version 25.0. Measurement of continuous normal distribution was expressed as mean ± standard deviation. *T* test was used for inter-group comparisons. Categorical variables were expressed as percentage and compared by chi-square test. Multinomial logistic regression was applied to predict the probability of different factors influencing the degree of disease. Statistical significance was defined as *p* value < 0.05.

## Results

### Comparison of demographic data and clinical characteristics of patients

In total, 394 patients were enrolled in this study. Among all of them, there were 305 mild cases including 173 males and 132 females aged from 10 month to 80 years (33.09 ± 1.012 years). These patients have various underlying diseases or medical conditions. The underlying diseases were categorized into cardiovascular and cerebrovascular diseases (hypertension, diabetes, cerebral infarction, coronary heart disease, vertigo, hyperlipidemia), blood diseases (anemia), digestive system diseases (chronic hepatitis B, gastritis), respiratory diseases (pulmonary tuberculosis, asthma, emphysema, sleep apnea syndrome, bronchiectasis), gynecological diseases (hysteromyoma, teratoma), ophthalmic diseases (glaucoma, cataract), allergic conditions (rhinitis, urticaria), kidney diseases (renal insufficiency), Rheumatoid arthritis and autoimmune diseases (ankylosing spondylitis), endocrine disease (hyperthyroidism), and tumor (uterine fibroids and ductal carcinoma in situ, poorly differentiated adenocarcinoma of the upper lobe of the left lung with multiple metastases to bone, lymph nodes, left pleura and abdominal wall, thyroid cancer) The incident rates of the disease categories in the disease groups are summarized in Table 1. There are in total 8 cases of hypertension; 3 cases of diabetes; 2 cases of obsolete pulmonary tuberculosis; 2 cases of renal insufficiency; 2 cases of pregnancy; 2 cases of coronary heart disease, 1 case of cataract, glaucoma, chronic hepatitis B, anemia, ankylosing spondylitis, cerebral infarction, uterine fibroids and ductal carcinoma in situ respectively; and 1 case of poorly differentiated adenocarcinoma of the upper lobe of the left lung with multiple metastases to bone, lymph nodes, left pleura and abdominal wall and cardiac insufficiency.

**Table 1:** Comparison of underlying diseases of each group of patients

	Mild group	Moderate group	Total	*p* value
Number of patients	305	89	394	
With underlying disease	28 (9.18%)	36 (40.44%)	64 (16.24%)	0.0000
Without underlying disease	277 (90.8%)	53 (59.55%)	330 (83.76%)	0.0000
Cardiovascular and cerebrovascular diseases	14 (4.6%)	14 (15.7%)	28 (7.1%)	0.0003
Digestive system diseases	1 (0.3%)	2 (2.2%)	3 (0.8%)	0.0668
Respiratory diseases	3 (1.0%)	5 (5.6%)	8 (2.0%)	0.0064
Gynecological diseases	1 (0.3%)	1 (1.1%)	2 (0.5%)	0.3527
Ophthalmic diseases	2 (0.6%)	1 (1.1%)	3 (0.8%)	0.6551
Allergic conditions	0 (0)	2 (2.2%)	2 (0.5%)	0.0087
Kidney diseases	2 (0.6%)	0 (0)	2 (0.5%)	0.4437
Rheumatoid arthritis and autoimmune diseases	1 (0.3%)	0 (0)	1 (0.3%)	0.5886
Tumor	2 (0.6%)	1 (1.1%)	3 (0.8%)	0.6551
Blood diseases	1 (0.3%)	3 (3.4%)	4 (1.0%)	0.0118
Endocrine system diseases	0 (0)	1 (1.1%)	1 (0.3%)	0.0638

There were 89 moderate cases, among which 50 are males and 39 are females, aged from 1 month to 91 years (38.97 years ± 1.936 years). Underlying diseases of these patients include 6 cases of hypertension; 4 cases of diabetes; 3 cases of hyperthyroidism, chronic hepatitis B and anemia respectively; 2 cases of emphysema, cerebral infarction and asthma, respectively; 1 case of thyroid cancer (postoperative), vertigo, rhinitis and gastritis, urticaria, sleep apnea, hyperlipidemia, pulmonary tuberculosis, teratoma, bronchiectasis and glaucoma, respectively.

Results of Chi-square test of all diseases groups gave a *χ*^2^ of 49.5135 and a *p* value of 0.00. Among two disease groups, mild group has lower number of underlying diseases and lower ratio of patients with underlying diseases. In comparison, moderate group has higher ratio of patients with higher number of underlying diseases, suggesting an association between underlying diseases and disease severity of Omicron infection.

There are 106 asymptomatic cases among all patients. Clinical symptoms of most of patients were fever, sore throat, cough, expectoration, stuffy and runny nose and fatigue. Comparing to mild group, significantly higher ratio of patients in the moderate group had symptoms including fever, dry throat/itchy throat/sore throat, diarrhea, body ache and soreness, stuffy and runny nose (Table 2).

**Table 2:** Comparison of clinical symptoms of each group of patients

Symptoms	Mild group		Moderate group			Significance
	No	Ratio (%)	No.	Ratio (%)	*χ*2	*p* value
Fever	68	22.3	33	37.1	7.8949	0.0049
Dry throat/itchy throat/ sore throat	58	19.02	41	46.1	26.7976	0
Diarrhea	3	0.98	4	4.5	4.8661	0.0274
Chest tightness	7	2.3	3	3.4	0.0341	0.8535
Body ache and soreness	7	2.3	11	12.4	13.7817	0.0002
Stuffy and runny nose	29	9.5	16	18.0	4.0835	0.04
conjunctivitis	1	0.33	0	0	0.4308	0.5116
Fatigue	26	8.52	13	14.6	2.2164	0.1366
Loss of taste and smell	2	0.66	3	3.4	2.1761	0.1402
Shortness of breath	2	0.66	1	1.1	0.0606	0.8055
Nausea and vomiting	3	0.98	0	0	0.0606	0.8055
Cough and expectoration	142	46.56	70	78.7	27.2752	0

### Summary of immunization status showed that no difference in vaccination rate between disease groups

We then summarized the vaccination rate of all patients to discover the protective effect of vaccine against Omicron infection. Among all, 64 patients were not vaccinated while 330 of them were vaccinated with different numbers of doses, including 30 with one dose, 156 with two doses, 139 with three doses and 5 with four doses (Table 3). In the mild group, 49 cases were not vaccinated and 305 cases were vaccinated, among which 199 patients received inactivated viral vaccine, 51 received mRNA vaccine and 5 received viral vector vaccine. 29 of them received one dose, 116 received 2 doses, 108 received 3 doses and 3 received 4 doses. For the 89 patients in the moderate group, 15 cases were unvaccinated while 74 cases were vaccinated. Among immunized patients, 1 of them received 1 dose, 40 cases received 2 doses, 31 cases received 3 doses and 2 cases received 4 doses. 67 patients were immunized with inactivated viral vaccine and 7 patients were immunized with mRNA vaccine. There was no significant difference in vaccination rate observed between the two disease groups.

**Table 3:** Vaccination rates of patients in different groups

Group	Mild (%)	Moderate (%)	Total	*P*
Number of patients	305 (77.41)	89 (22.59)	394	–
Nonvaccinated	49 (16.07)	15 (16.85)	64 (16.24)	0.8592
Vaccinated	256 (83.93)	74 (84.27)	330 (83.76)	0.8592
One dose	29 (9.51)	1 (1.12)	30 (7.61)	0.0087
Two doses	116 (38.03)	40 (44.94)	156 (39.59)	0.2408
Three doses	108 (35.41)	31 (34.83)	139 (35.28)	0.92
Four doses	3 (0.98)	2 (2.25)	5 (1.27)	0.3488
Inactivated Viral vaccine	199 (65.25)	67 (75.28)	266 (67.51)	0.0753
mRNA vaccine	51 (16.72)	7 (7.87)	58 (14.72)	0.0038
viral vector vaccine	5 (1.64)	0 (0)	5 (1.27)	0.2241
Recombinant subunit vaccine	1 (0.33)	0 (0)	1 (0.25)	0.5886

Percentages were calculated by dividing the number of patients in each category by the number of patients in each disease group

### Laboratory examination indicated that mild group has higher IgG level and lower IL-6 level

**Fig. 1:**
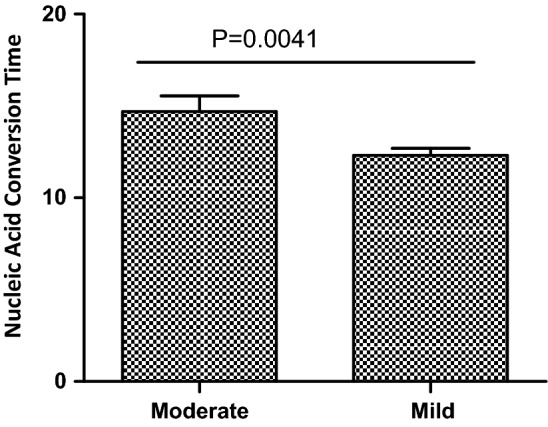
Shorter nucleic acid conversion time observed from the mild group

Results of blood examinations of patients in different disease groups were analyzed and compared to figure out the difference in patients’ responses to Omicron infection. As shown in Table 4, levels of IgG and lactate dehydrogenase (LDH) were significantly higher (*p* value < 0.05) in the mild group. Levels of interleukin-6 (IL-6), erythrocyte sedimentation rate (ESR), fibrinogen (FIB) were significantly higher in the moderate group. Levels of urea, creatinine, glucose, troponin I (Trop I), myoglobin and creatinine kinase isoenzymes (CK-MB) were also significant different between the two disease groups. Levels of PCT, IL-6, C-reactive protein (CRP), IgG, and Trop I were all beyond their normal ranges while level of pALB was under its normal range in the two disease groups. Levels of urea and CK-MB were beyond the normal ranges in the moderate group (Table 4). As IL-6 is associated with inflammation, such observation may infer that moderate group has more active immune responses comparing to mild group. We also observed that nucleic acid conversion times (time taken for nucleic acid PCR result converting from positive to negative) were 14.70 ± 0.8428d for the moderate group and 12.31 ± 0.3718d for the mild group (Fig. 1). Significantly shorter nucleic acid conversion time was observed from the mild group.

**Table 4:** Analysis of differences of blood indexes among different disease groups

Indicator (range)	Level in Moderate group (mean/% above upper limit)	Level in Mild group (mean/% above upper limit)	*p*-value
Number of patients	*n* = 89	*n* = 305	
Procalcitonin (PCT, < 0.1 ng/ml)	0.204 (78.65)	0.196 (82.95)	0.5516
Interleukin-6 (IL-6, 0-7 pg/mL)	20.097 (78.65)	13.88 (69.84)	0.0098
C-reactive protein (CRP, 0-4 mg/L)	7.752 (51.7)	5.84 (38.36)	0.0625
Erythrocyte sedimentation rate (ESR30, 0-20 mm/H)	16.045 (24.72)	10.19 (9.18)	0.0004
Prothrombin time (PT, 11–15.1 s)	13.401 (3.4)	13.44 (2.30)	0.7289
Activated partial thromboplastin time (APTT, 28–43.5 s)	39.809 (15.73)	38.87 (13.77)	0.1333
Fibrinogen (FIB, 2-4 g/L)	3.279 (15.7)	3.04 (18.52)	0.0038
D-dimer (D-DIC, 0–0.5ug/mL)	0.623 (11.24)	0.39 (12.46)	0.0816
White blood cell (WBC,3.5–9.5 × 10^9^/L)	5.992 (2.2)	6.20 (5.9)	0.3883
Neutrophil percentage (NEUT%,40–75%)	64.924 (21.3)	64.07 (22.30)	0.6116
Eosinophil percentage (EO%, 0.4–8%)	1.616 (3.4)	1.59 (2.62)	0.9175
Lymphocyte count (LYMPH#,1.1–3.2 × 10^9^/L)	1.356 (3.4)	1.47 (3.93)	0.2769
Hemoglobin (HGB, 115-150 g/L)	137.36 (23.6)	141.36 ( 32.79)	0.0845
Platelet (PLT, 99–303 × 10^9^/L)	226.708 (12.4)	229.17 (13.44)	0.765
ORFLAB Ct value	23.23 (13.08–39.33)	23.97 (13.38–39.82)	0.3957
NJY Ct value	23.045 (12.21–39.09)	23.55 (12.01–39.03)	0.577
SARS-CoV-2-IgM (0-10AU/mL)	1.293 (2.2)	1.55 (2.62)	0.8067
SARS-CoV-2-IgG (0-10AU/mL)	87.655 (59.6)	135.41 (65.9)	0.0079
Sodium (Na + , 135–145 mmol/L)	140.347 (4.5)	140.86 (7.21)	0.1781
Potassium (K + , 3.5–5.1 mmol/L)	3.664 (2.25)	3.76 (0.66)	0.0634
Chlorine (CL-, 98-107 mmol/L)	104.835 (15.7)	105.32 (19.67)	0.1427
Phosphate (PHOS, 0.81–1.45 mmol/L)	1.281 (11.2)	1.33 (6.56)	0.1755
Albumin (ALB, 35-50 g/L)	45.164 (10.1)	45.28 (8.85)	0.7999
Prealbumin (pALB, 200.0–400.0 mg/L)	192.716 (30–339)	203.75 (34–323)	0.0769
Total bilirubin (TBIL, 3.0-22umol/L)	12.808 (5.6)	12.97 (6.56)	0.8348
(Bu, 0-19umol/L)	9.737 (5.6)	9.50 (5.57)	0.7671
δ-bilirubin (δBIL, 0-3umol/L)	3.071 (44.94)	3.47 (51.15)	0.1164
Alanine aminotransferase (ALT, 0-50U/L)	29.195 (11.2)	28.74 (11.15)	0.8989
Aspartate aminotransferase (AST, 17-59U/L)	31.633 (3.4)	32.40 (3.93)	0.6957
Lactate dehydrogenase (LDH, 120-246U/L)	153.759 (5.6)	176.10 (3.93)	0.0161
Creatinine (CREA, 58-110umol/L)	53.526 (3.4)	66.36 (1.31)	< 0.0001
Urea (3.2–7.1 mmol/L)	19.686 (12.45)	6.29 (6.23)	< 0.0001
Glucose (4.1-11 mmol/L)	4.632 (1.1)	5.46 (1.64)	0.0027
Troponin I (Trop I, < 0.034, AMI threshold = 0.12ug/L)	18.81 (25.8)	1.72 (4.92)	< 0.0001
Myoglobin (0-121 ng/mL)	50.861 (6.7)	32.97 (2.30)	0.028
N-terminal pro-brain natriuretic peptide (NT-proBNP, < 75 yrs: 0–125; > 75 yrs: 0-450 pg/mL)	64.838 (3.4)	89.23 (2.30)	0.7045
Creatinine kinase isoenzymes (CK-MB, 0–2.37 ng/mL)	11.538 (27.0)	2.11 (6.89)	< 0.0001

^a^p value < 0.05

The probability of disease deterioration is higher if the patient has underlying diseases. There was no significant statistical difference between genders, the number of vaccine injection on the severity of symptoms. However, IgG level and underlying diseases are factors influencing the severity of symptoms. Patients with underlying diseases have 4.6 times higher probability to progress to more severe symptoms comparing to those without underlying diseases (Table 5).

**Table 5:** Logistic regression estimates of patients by their characteristics

N = 392, Mild = 303; Moderate = 89						
	Odds ratio	OR (95% CI)		AIC	BIC	Pearson chi2
Age	1.011	0.996	1.026	400.663	− 1896.388	394.000
Male	Ref.					
Female	1.109	0.667	1.845			
Number_of_vaccinations	1.333	0.847	2.097			
IgM	1.014	0.979	1.051			
IgG	0.997^b^	0.994	0.999			
Time_internal [1]	1.004^a^	1.000	1.007			
No underlying diseases	Ref.					
With underlying disease	4.633^c^	2.251	9.537			
No vaccination	Ref.					
Vaccine category (inactivated virus)	0.554	0.172	1.785			
Vaccine category (mRNA)	0.460	0.123	1.727			
Vaccine category (others)	0.889	0.166	4.751			

[1] The interval between the last blood collection and the date of admission in hospital

*OR* Odd ratio; *CI* Confidence interval

^a^p < 0.05

^b^p < 0.01

^c^p < 0.001

## Discussion

Based on the clinical classification of the patients infected with Omicron involved in this study, the majority of them have mild or moderate symptoms. There were no severe or death cases occurred during this study due to the decreased pathogenicity of Omicron variant. The clinical manifestations of patients infected with Omicron variant are mainly upper respiratory symptoms, including fever, sore throat, cough, expectoration, nasal congestion, runny nose and fatigue. The clinical symptoms caused by Omicron variant are milder than those caused by Delta variant. This is consistent with what has been reported by the study group from South Africa [4]. In our study, we found that the proportion of symptoms such as fever, dry throat/itchy throat/sore throat, diarrhea, body ache and soreness and stuffy and runny nose in moderate group is higher than that of mild group. Omicron variant is often confined to the upper respiratory tract like the nose, the throat and the respiratory tract, which causes less damage to the lungs comparing to other variants. The reason may be that many cells carry protein named TMPRSS2 on the cell surfaces, which helps the internalization of virus to the cells in the lungs [5]. However, omicron variant displayed less fusion and replication in cells with TMPRSS2 protein [6]. In such way, Omicron has lower pathogenicity to the lung cells comparing to Delta variant [7]. Although with reduced fatality rate as compared to Delta variant, Omicron variant can still cause death of patients [8]. In addition to physical protection measure taken to prevent the spread of COVID-19 such as wearing medical masks, quarantine and isolation, COVID-19 vaccine is another important and effective measure to control this pandemic [9]. Previous studies have indicated that vaccination is effective against severe or fatal cases of Omicron infection [10, 11]. Different countries around the world are developing various types of COVID-19 vaccines, including nucleic acid vaccine, inactivated viral vaccine, live attenuated vaccine, viral vector vaccine, subunit vaccine, recombinant protein vaccine and etc. [12]. There are already five types of COVID-19 vaccines released in China, including adenovirus vector vaccine (one injection), inactivated viral vaccine (two injections) and recombinant protein vaccine (three injections). Among them, inactivated viral vaccines, coronavac (Sinovac Life Sciences, Beijing, China) and bbibp CorV (Beijing Institute of biological products, Beijing, China) are the most frequently vaccinated types in China. Many studies have demonstrated the efficacy of inactivated viral vaccines [13–16]. In our study, although no significant difference was observed from the vaccination rate between the two disease groups, over 80% of the patients were vaccinated and none of them was severe or critically ill, showing that the vaccines would help in the prevention of disease progress and aggravation from mild/moderate to severe illness. However, even with the protection of vaccine, there are still considerable number of patients developed lower respiratory tract infection in moderate group. The levels of IL-6 in these patients were higher than those in mild group, suggesting a more activated immune system. This might be due to the higher rate of underlying diseases in moderate group since there was no difference in viral load and inflammatory index CRP level between the two groups. The nucleic acid conversion time of the moderate group was also longer as they needed more time to clear the virus. Our study also showed that patients with underlying diseases are more likely to develop lung infections (4.6 times higher probability). Previous studies have pointed out that 26% of the patients infected with Delta variant had higher blood glucose level at admission, whereas blood glucose level was an independent risk factor for COVID-19 progression to severe, critical illness or death [17]. Blood glucose level controlled within the range of 3.9 to 10 mmol/L could significantly reduce the incidence of serious adverse events and mortality in patients with COVID-19 combined with diabetes [18].

In conclusion, in terms of control and prevention of COVID-19, immunization by vaccination should be promoted and optimized among elderly population with underlying diseases to prevent disease progress to severe and critical illness. In clinical perspective, more attention should be paid to such vulnerable population and prevent aggravation of COVID-19. Owing to the limitation in the number of patients involved, we cannot make statistical analysis on the types of underlying diseases among different disease groups. Further study involving a larger sample size is needed for correlation verification. This study gave an insight into the clinical and epidemiological characteristics of Omicron variant infection and provided a scientific basis for the prevention and control of such infection.

## Data Availability

All data generated or analyzed during this study are included in this published article.
